# The prognostic value of CYP2C subfamily genes in hepatocellular carcinoma

**DOI:** 10.1002/cam4.1299

**Published:** 2018-02-26

**Authors:** Xiangkun Wang, Tingdong Yu, Xiwen Liao, Chengkun Yang, Chuangye Han, Guangzhi Zhu, Ketuan Huang, Long Yu, Wei Qin, Hao Su, Xiaoguang Liu, Tao Peng

**Affiliations:** ^1^ Department of Hepatobiliary Surgery The First Affiliated Hospital of Guangxi Medical University Nanning 530021 Guangxi Province China; ^2^ Department of Hepatobiliary and Pancreatic Surgery The First Affiliated Hospital of Zhengzhou University Zhengzhou Henan Province 450000 China; ^3^ Department of Hepatobiliary Surgery Affiliated Hospital of Guangdong Medical University Zhanjiang Guangdong Province 524001 China

**Keywords:** CYP2C subfamily, gene expression, hepatocellular carcinoma, prognosis, serum biomarker

## Abstract

Cytochrome P2C (CYP2C) subfamily members (*CYP2C8*,*CYP2C9*,*CYP2C18*, and *CYP2C19*) are known to participate in clinical drug metabolism. However, the association between CYP2C subfamily members and hepatocellular carcinoma (HCC) remains unclear. This study investigated the prognostic value of CYP2C subfamily gene expression levels with HCC prognosis. Data of 360 HCC patients in The Cancer Genome Atlas database and 231 in the Gene Expression Omnibus database were analyzed. Kaplan–Meier analysis and a Cox regression model were used to ascertain overall survival and recurrence‐free survival, and to calculate median survival time using hazard ratios (HR) and 95% confidence intervals (CI). In TCGA database, low expression of *CYP2C8*,*CYP2C9*, and *CYP2C19* in tumor tissue was associated with a short median survival time (all crude *P *= 0.001, adjusted *P *= 0.004, *P *= 0.047, and *P *= 0.020, respectively). In TCGA database, joint effects analysis of the combinations of *CYP2C8* and *CYP2C9*,*CYP2C8* and *CYP2C19*, and *CYP2C9* and *CYP2C19* revealed that high expression of two genes (group 4; group IV, group **d**) was associated with a reduced risk of death as compared to low expression (group 1, group I, and group **a**) (adjusted *P* = 0.005, *P* = 0.013, and *P* = 0.016, respectively). In TCGA database, joint effects analysis of *CYP2C8*,*CYP2C9*, and *CYP2C19* showed that the risk of death from HCC was lower for groups C and D than for group A (adjusted *P* = 0.012 and *P* = 0.008, respectively). *CYP2C8*,*CYP2C9*, and *CYP2C19* gene expression levels are potential prognostic markers of HCC following hepatectomy.

## Introduction

Liver cancer is the fifth most commonly diagnosed cancer and the second most frequent cause of cancer‐related deaths in men and the seventh most frequently diagnosed cancer and the sixth leading cause of cancer‐related deaths in women worldwide 1. There were about 4,292,000 newly diagnosed cases and 2,814,000 deaths from cancer in China in 2015 2. Hepatocellular carcinoma (HCC), the major histological type, accounts for most (70–85%) cases of primary liver cancer worldwide 3. Etiologically, infection of hepatitis C or B virus (HBV), aflatoxin exposure, obesity, diabetes, nonalcoholic steatohepatitis, alcohol ingestion, hemochromatosis, and other metabolic diseases are the primary risk factors for HCC 4. Despite advances in several treatment strategies, such as liver resection, liver transplantation, percutaneous ethanol injection, transcatheter arterial chemoembolization, transarterial radiation, microwave ablation, and systemic therapy, the prognosis of HCC remains unsatisfactory because of late‐stage diagnosis 5, which has resulted in a reported 5‐year survival rate of only 7% 6. Thus, the identification of molecular biomarkers for the early diagnosis of HCC is crucial to provide more effective therapies and improve patient prognosis.

Cytochrome P2 (CYP2) family members of the CYP superfamily include many subfamilies, such as CYP2A, CYP2B, CYP2C, CYP2D, CYP2E, and CYP2F. The human CYP2C subfamily consists of four members (*CYP2C8*,* CYP2C9*,* CYP2C18*, and *CYP2C19*) that are localized in a single gene locus on chromosome 10 7, 8. Members of the CYP2C subfamily are known to be involved in the metabolism of roughly 20% of clinically used drugs, such as the anticancer drug paclitaxel 9, the antidiabetic agent tolbutamide 8, proton pump inhibitors 10, as well as various endogenous and exogenous substances 11. In addition, *CYP2C8* is reportedly related with an increased risk of essential hypertension and coronary artery disease in Bulgarians 12 and has also been associated with anemia 13, breast cancer 14, and vascular inflammatory diseases 15. Moreover, *CYP2C9* is reportedly associated with the risk of colorectal cancer 16, while *CYP2C18* was found to have no contribution to cancer risk 11 and *CYP2C19* has been associated with peptic ulcer disease 17, colorectal adenoma recurrence 18, breast cancer 19, and cardiovascular diseases 20. However, little is known about the associations of the expression levels of these four genes with the risk of HCC. Thus, the aim of this study was to identify relationships between CYP2C expression levels and HCC prognosis.

## Material and Methods

### Patient data

First, the Metabolic gEne RApid Visualizer database (http://merav.wi.mit.edu/) was accessed on September 10, 2017 to determine whether any of the four members of the CYP2C subfamily are differentially expressed between normal liver tissues and primary liver tumors. Then, the GTExPortal (https://gtexportal.org/home/) was accessed on September 10, 2017 to obtain gene expression levels of CYP2C subfamily in different tissues 21. Moreover, the Search Tool for the Retrieval of Interacting Genes/Proteins (STRING) database was accessed on September 10, 2017 to construct protein–protein interaction networks between CYP2C subfamily members and other proteins.

The OncoLnc (http://www.oncolnc.org/) and The Cancer Genome Atlas (TCGA), (http://tcga-data.nci.nih.gov/tcga) databases were accessed on September 10, 2017 to acquire data regarding the gene expression levels of *CYP2C8*,* CYP2C9*,* CYP2C18*, and *CYP2C19*, as well as the corresponding 50% cutoff values. The results presented here, in part, are based on TCGA studies 22. Data of 360 HCC patients, including sex, race, age, body mass index (BMI), tumor, node, metastasis (TNM) stage, survival time, and survival status, were collected. Gene expression data were downloaded from the GSE14520 dataset of the Gene Expression Omnibus (GEO) database (https://www.ncbi.nlm.nih.gov/geo/query/acc.cgi?acc=GSE14520) on September 12, 2017 23. The GSE14520 dataset included gene expression levels originated from [HT_HG–U133A] Affymetrix HT Human Genome U133A 23 and [HT_HG–U133A_2] Affymetrix HT Human Genome U133A_2.0 24 arrays. In order to prevent batch effects, the former array of 231 HCC patients (more patients than the latter, 445 samples) was chosen.

### Functional enrichment analysis of the CYP2C subfamily

The Database for Annotation, Visualization, and Integrated Discovery (DAVID) v.6.7 (https://david-d.ncifcrf.gov/) was accessed on September 15, 2017 25, 26 for enrichment analysis, gene ontology (GO) functional analysis, and Kyoto Encyclopedia of Genes and Genomes (KEGG) pathway analysis. GO analysis is composed of terms of biological processes (BP), cellular components (CC), and molecular functions (MF); in the latter, KEGG pathways were drawn between CYP2C and other subfamilies.

### Survival analysis

From the TCGA database, 360 HCC patients were divided into two groups of 180 patients each at a 50% cutoff value. The median survival time (MST) was applied to estimate patient prognosis and TNM stage in a Cox regression model adjusted for patient age and sex. In order to assure a rational comparison between the above two databases, the 50% cutoff was used for the GEO database. In the GEO database, overall survival (OS) and recurrence‐free survival (RFS) were applied to evaluate patient prognosis. In addition, the Cox regression model was adjusted for age, sex, alanine aminotransferase level, nodal status, HBV status, primary tumor size, alpha‐fetoprotein (AFP) level, cirrhosis status, and Barcelona Clinic Liver *Cancer* (BCLC) stage.

### Joint effects analysis of *CYP2C8*,* CYP2C9*, and *CYP2C19*

In the TCGA database, only *CYP2C8*,* CYP2C9*, and *CY*P2C19 were statistically significant. Joint effects analysis was conducted with the following combinations: (1) *CYP2C8* and *CYP2C9*; (2) *CYP2C8* and *CY*P2C19; (3) *CYP2C9* and *CY*P2C19; and (4) *CYP2C8*,* CYP2C9*, and *CY*P2C19.

Combinations of *CYP2C8* and *CYP2C9* were composed of four groups: group 1 (low *CYP2C8* and low *CYP2C9* expression), group 2 (low *CYP2C8* and high *CYP2C9* expression), group 3 (high *CYP2C8* and low *CYP2C9* expression), and group 4 (high *CYP2C8* and high *CYP2C9* expression).

Combinations of *CYP2C8* and *CY*P2C19 were composed of four groups: group I (low *CYP2C8* and low *CY*P2C19 expression), group II (low *CYP2C8* and high *CY*P2C19 expression), group III (high *CYP2C8* and low *CY*P2C19 expression), and group IV (high *CYP2C8* and high *CY*P2C19 expression).

Combinations of *CYP2C9* and *CY*P2C19 were composed of four groups: group **a** (low *CYP2C9* and low *CYP2C19* expression), group **b** (low *CYP2C9* and high *CYP2C19* expression), group **c** (high *CYP2C9* and low *CYP2C19* expressions), and group **d** (high *CYP2C9* and high *CYP2C19* expression).

Combinations of *CYP2C8*,* CYP2C9*, and *CY*P2C19 were composed of four groups: group A (low *CYP2C8*, low *CYP2C9*, and low *CYP2C19* expression); group B (high *CYP2C8*, low *CYP2C9*, and low *CYP2C19* expression; low *CYP2C8*, high *CYP2C9*, and low *CYP2C19* expression; and low *CYP2C8*, low *CYP2C9*, and high *CYP2C19* expression); group C (high *CYP2C8*, high *CYP2C9*, and low *CYP2C19* expression; high *CYP2C8*, low *CYP2C9*, and high *CYP2C19* expression; and low *CYP2C8*, high *CYP2C9*, and high *CYP2C19* expression); and group D (high *CYP2C8*, high *CYP2C9*, and high *CYP2C19* expression). The Cox regression model was adjusted for TNM stage, age, and sex in keeping with the above combinations.

### Statistical analysis

The Pearson correlation coefficient was used to assess correlations among the *CYP2C8*,* CYP2C9, CYP2C18*, and *CYP2C19* genes. Correlation plots were depicted by R v.3.2.0 (https://www.r-project.org/). Interactions among these four genes and others as well as the four proteins encoded by these with others were drawn with the Cytoscape v.3.5.1 open source software platform for visualizing complex networks (http://www.cytoscape.org/). MST and probability (*P*) values were calculated by Kaplan–Meier survival analysis and the log‐rank test. Univariate and multivariate survival analysis were performed using the Cox hazards regression model. Scatter diagrams and survival curves were constructed using GraphPad Prism v.7 software (GraphPad Software, Inc., La Jolla, CA). All statistical analyses were performed using SPSS v.16 software (SPSS, Inc., Chicago, IL, USA). A *P* < 0.05 was considered statistically significant.

## Results

### Basic patient data

Detailed characteristics of the 360 patients in the TCGA database are shown in Table 1. TNM stage was significantly associated with MST (*P* < 0.001), but not sex, age, BMI, or race (all *P *> 0.05).

**Table 1:** Basic characteristics of 360 HCC patients

Variables	Patients (*n* = 360)	No. of events (%)	MST (days)	HR (95% CI)	Log‐rank *P* value
Race					
Asian	155	44 (28.4%)	NA	Ref.	0.185
White + others	196	78 (39.8%)	1397	1.29 (0.89–1.87)	
Missing^Đ^	9				
Sex					
Male	244	78 (32.0%)	2486	Ref.	0.309
Female	116	48 (41.4%)	1560	1.21 (0.84–1.73)	
Age(year)					
<60	168	54 (32.1%)	2532	Ref.	0.363
≥60	189	70 (37.0%)	1685	1.18 (0.83–1.68)	
Missing^†^	3				
BMI					
≤25	193	66 (34.2%)	2456	Ref.	0.478
>25	137	45 (32.8%)	2116	0.87 (0.60–1.27)	
Missing^ý^	30				
TNM stage					
A + B	252	66 (26.2%)	2532	Ref.	**<0.001**
C + D	87	48 (55.2%)	770	2.50 (1.72–3.63)	
Missing^Ĺ^	21				

BMI, body mass index; TNM stage, tumor, node and metastasis stage; MST, median survival time; HR, hazard ratio; 95% CI, 95% confidence interval; Ref, reference; Missing^Đ^, information of race was unavailable in 9 patients; Missing^†^, information of age was unavailable in 3 patients; Missing^ý^, information of BMI was unavailable in 30 patients; Missing^Ĺ^, information of TNM stage was unavailable in 21 patients. The significance is that all the values are statistically significant.

The data of the 231 patients from the GEO database are presented in Table 2. Sex, nodal status, primary tumor size, BCLC stage, cirrhosis status, and AFP level were related to OS (all *P* = 0.048, 0.003, <0.001, <0.001, 0.004, and 0.001, respectively), while sex, cirrhosis status, primary tumor size, and BCLC stage were related to RFS (*P* = 0.001, 0.019, 0.020, and <0.001, respectively).

**Table 2:** Basic characteristics of 231 HCC patients

Variables	Patients (*n* = 231)	Overall survival			Recurrence‐free survival		
		MST (months)	HR (95% CI)	Log‐rank *P*	MST (months)	HR (95% CI)	Log‐rank *P*
Sex							
Male	191	NA	Ref.	**0.048**	40	Ref.	**0.001**
Female	30	NA	0.59 (0.34–1.00)		NA	0.47 (0.29–0.75)	
Missing^Ʒ^	10						
Age							
≤60	181	NA	Ref.	0.852	46	Ref.	0.937
>60	40	NA	0.96 (0.65–1.44)		37	1.01 (0.73–1.41)	
Missing^Ʒ^	10						
HBV–virus status							
AVR–CC	56	NA	Ref.	0.149	30	Ref.	0.092
CC + NO	162	NA	0.78 (0.56–1.09)		48	0.78 (0.59–1.04)	
Missing^ƛ^	13						
ALT							
≤50 U/L	130	NA	Ref.	0.710	53	Ref.	0.090
>50 U/L	91	NA	1.06 (0.78–1.44)		40	1.25 (0.97–1.61)	
Missing^Ʒ^	10						
Main tumor size							
≤5 cm	140	NA	Ref.	**<0.001**	51	Ref.	**0.020**
>5 cm	80	53	1.87 (1.38–2.55)		30	1.37 (1.05–1.78)	
Missing^ƥ^	11						
Multinodular							
Yes	45	48	Ref.	**0.003**	27	Ref.	0.136
No	176	NA	0.59 (0.42–0.84)		49	0.79 (0.58–1.08)	
Missing^Ʒ^	10						
Cirrhosis							
Yes	203	NA	Ref.	**0.004**	38	Ref.	**0.019**
No	18	NA	0.23 (0.09–0.63)		NA	0.50 (0.28–0.89)	
Missing^Ʒ^	10						
BCLC stage							
0+A	168	NA	Ref.	**<0.001**	58	Ref.	**<0.001**
B+C	51	20	3.63 (2.64–5.00)		18	2.84 (2.14–3.75)	
Missing^Ɯ^	12						
AFP							
≤300 ng/ml	100	NA	Ref.	**0.001**	49	Ref.	0.094
>300 ng/ml	118	NA	1.67 (1.23–2.27)		31	1.24 (0.96–1.61)	
Missing^ƛ^	13						

AVR–CC, active viral replication chronic carrier; CC, chronic carrier; ALT, alanine aminotransferase; AFP, alpha fetoprotein; BCLC stage, Barcelona Clinic Liver Cancer; Missing^Ʒ^, information of sex, age, ALT, multinodular, cirrhosis was unavailable in 10 patients; Missing^ƥ^, information of main tumor size was unavailable in 11 patients; Missing^Ɯ^, information of BCLC stage was unavailable in 12 patients; Missing^ƛ^, information of HBV–virus status and AFP was unavailable in 13 patients. The significance is that all the values are statistically significant.

### Analysis of CYP2C subfamily gene expression levels in tumor and nontumor tissues

**Figure 1:**
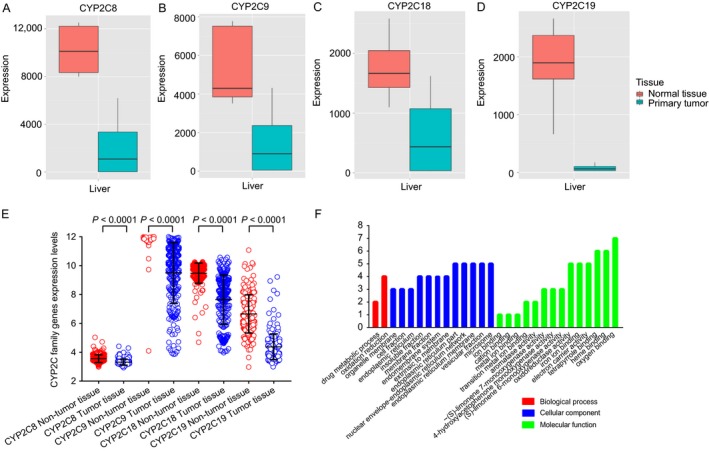
Gene expression levels of *CYP2C8* (A), *CYP2C9* (B), *CYP2C18* (C), and *CYP2C19* (D) in normal liver tissue and primary liver tumors. Expression levels in the GEO database (E) and GO analysis (F) of the four genes.

Expression levels of *CYP2C8*,* CYP2C9, CYP2C18*, and *CYP2C19* in different organs are exhibited in the supplementary material. Box diagrams of the gene expression levels of *CYP2C8*,* CYP2C9, CYP2C18*, and *CYP2C19* were downloaded from an online website (Fig. 1A–D, respectively). The expression levels of these genes were high in normal liver tissues, but low in primary liver tumors. Scatter diagrams of these four genes from the GEO database showed that all generated statistically significant results between tumor and nontumor tissues (all *P* < 0.0001, Fig. 1E).

### Analysis of the GO and KEGG pathways of the CYP2C subfamily

**Figure 2:**
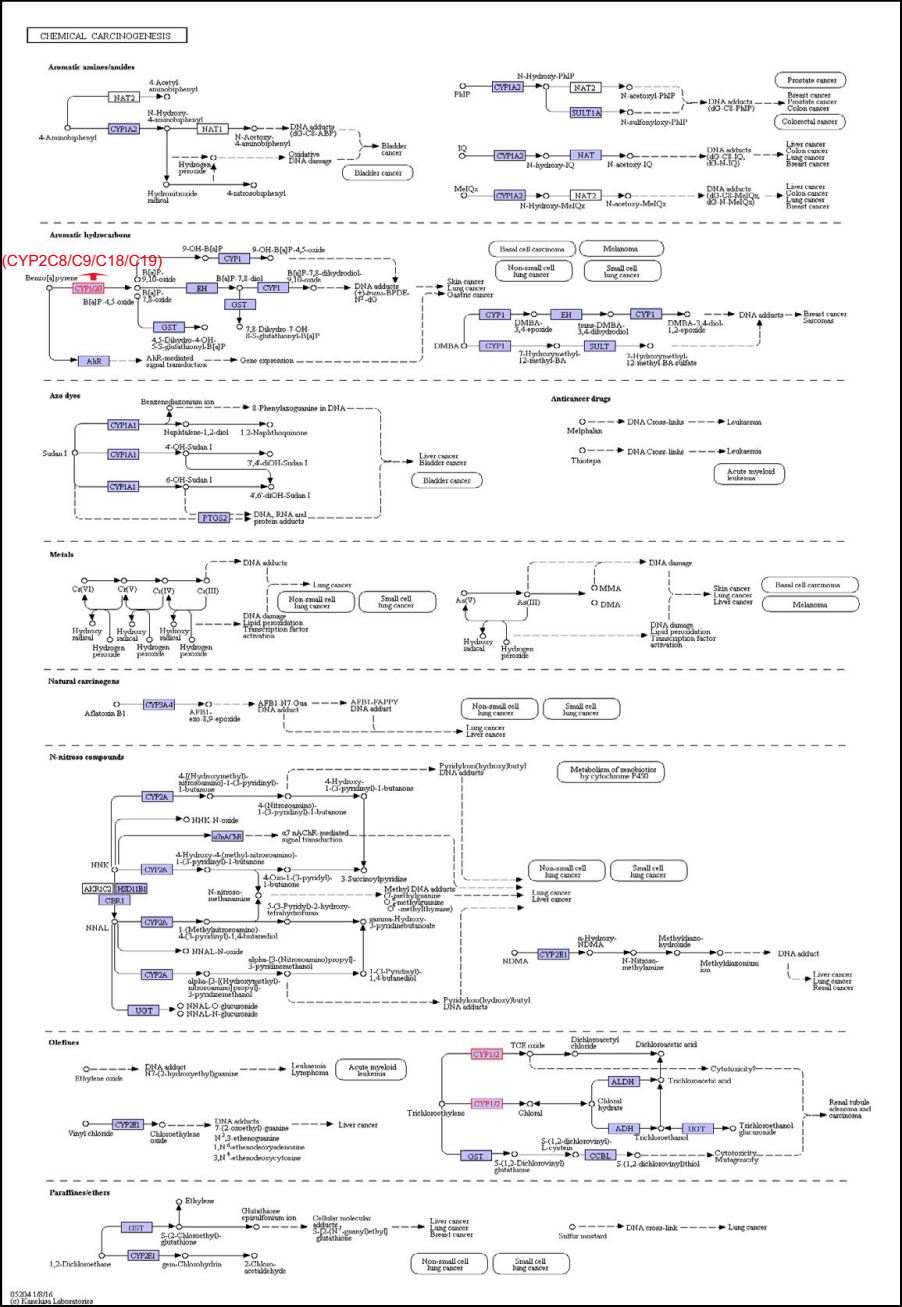
Metabolic pathways of the *CYP2C8*,*CYP2C9*,*CYP2C18*, and *CYP2C19* genes in chemical carcinogenesis.

The biological functions (BP, CC, and MF) of *CYP2C8*,* CYP2C9*,* CYP2C18*, and *CYP2C19* were evaluated using GO analysis, which showed that each were involved in drug metabolism and oxidation–reduction reactions. Detailed outcomes are shown in Figure 1F. In the KEGG pathway analysis, DAVID determined associations between CYP2C subfamily members and other genes. Benzo[a]pyrene can be metabolized by CYP2C subfamily members and finally transformed into DNA adducts, including (+)‐trans‐benzo[a]pyrene‐7, 8‐dihydrodiol‐9, and 10‐oxide (BPDE)‐N_2_‐dG, which are known to induce cancers of the skin, lung, and stomach (Fig. 2).

### Correlation analysis of the expression levels among CYP2C subfamily members

**Figure 3:**
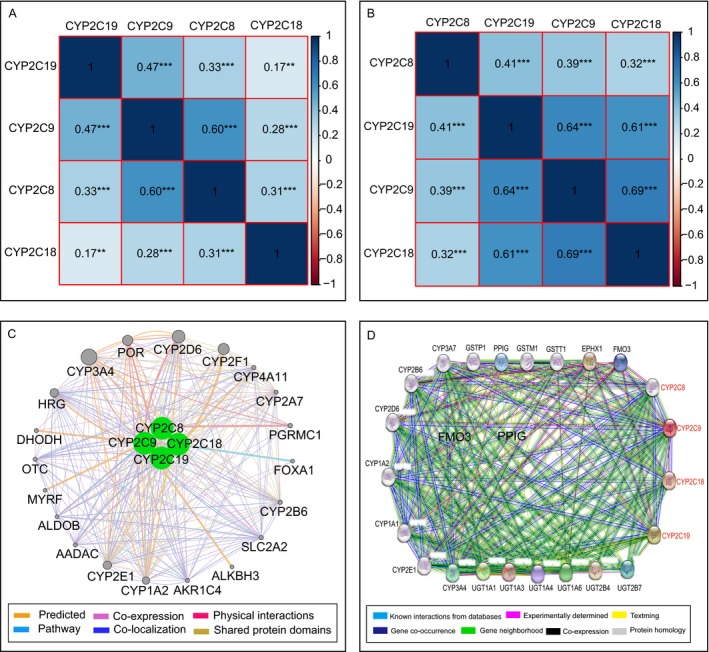
Matrix graphs of Pearson correlations of *CYP2C8*,*CYP2C9*,*CYP2C18*, and *CYP2C19* gene expression levels in the TCGA database (A) and GEO database (B). Gene–gene interaction networks among the four genes of interest with other genes (C) and protein–protein interaction networks among the four proteins of interest with other proteins (D).

The Pearson correlation coefficients of the four CYP2C members were calculated. In the TCGA database, each of these four genes was positively and significantly correlated with the other three members (all *P* < 0.05) (Fig. 3A). In the GEO database, all four genes were positively and statistically significantly correlated with the other three genes as well (all *P* < 0.05) (Fig. 3B).

Analysis of gene–gene interactions between CYP2C subfamily and other genes showed that these four genes were associated with other CYP subfamily members (*CYP1A2*,* CYP2A7*,* CYP2B6*,* CYP2D6*,* CYP2E1*,* CYP2F1*,* CYP3A4*, and *CYP4A11*) and other genes (*ALDOB*,* OTC*,* SLC2A2*,* PGRMC1*,* FOXC1*, etc.) (Fig. 3C). Moreover, protein–protein interaction networks were constructed using STRING database, which showed that the CYP family member proteins CYP1A1, CYP1A2, CYP2B6, CYP2D6, CYP2E1, CYP3A4, and CYP3A7 were also associated with CYP2C8, CYP2C9, CYP2C18, and CYP2C19 (Fig. 3D).

### Survival analysis of CYP2C subfamily members

The prognostic‐related characteristics in the TCGA database of age, TNM stage, and sex were analyzed using a multivariate Cox regression model, which showed that *CYP2C8*,* CYP2C9*, and *CYP2C19* exhibited significant relationships with MST (adjusted *P* = 0.004, hazard ratio (HR) = 0.57, 95% confidence interval (CI) = 0.39–0.84; adjusted *P* = 0.047, HR = 0.67, 95% CI = 0.46–1.00; and adjusted *P* = 0.020, HR = 0.63, 95% CI = 0.43–0.93, respectively, Table 3). In the GEO database, sex, age, HBV status, alanine aminotransferase level, primary tumor size, nodal status, BCLC stage, AFP level, and cirrhosis status were analyzed using a multivariate Cox regression model, which showed that *CYP2C8*,* CYP2C9, CYP2C18*, and *CYP2C19* were not statistically associated with OS or RFS (all *P* > 0.05, Table 4).

**Table 3:** Prognostic survival analysis of *CYP2C8, CYP2C9, CYP2C18* and *CYP2C19* genes in TCGA databases

Gene	Patients (*n *= 360)	MST (days)	Crude HR (95% CI)	Crude *P* value	Adjusted HR (95% CI)^a^	Adjusted *P* value^a^
*CYP2C8*						
Low	180	1229	Ref.	**0.001**	Ref.	**0.004**
High	180	2456	0.56 (0.39–0.79)		0.57 (0.39–0.84)	
*CYP2C9*						
Low	180	1271	Ref.	**0.001**	Ref.	**0.047**
High	180	2456	0.56 (0.39–0.80)		0.67 (0.46–1.00)	
*CYP2C18*						
Low	180	2456	Ref.	0.794	Ref.	0.845
High	180	1560	0.95(0.67–1.35)		0.96(0.66–1.40)	
*CYP2C19*						
Low	180	1229	Ref.		Ref.	
High	180	2456	0.55 (0.38–0.78)	**0.001**	0.63 (0.43–0.93)	**0.020**

a Adjusted *P*, adjustment for sex, age, TNM stage; *CYP2C8*, cytochrome P450 family 2 subfamily C member 8; *CYP2C9*, cytochrome P450 family 2 subfamily C member 9; *CYP2C18*, cytochrome P450 family 2 subfamily C member 18; *CYP2C19*, cytochrome P450 family 2 subfamily C member 19. The significance is that all the values are statistically significant.

**Table 4:** Prognostic survival analysis of *CYP2C8, CYP2C9, CYP2C18* and *CYP2C19* genes in GEO databases

Gene	Samples (*n* = 445)	Overall survival				Recurrence‐free survival			
		Crude HR (95% CI)	Crude *P* value	Adjusted HR(95% CI)	Adjusted *P* value	Crude HR (95% CI)	Crude *P* value	Adjusted HR (95% CI)^a^	Adjusted *P* value^a^
*CYP2C8*									
Low	223	Ref.	0.415	Ref.	0.721	Ref.	0.2	Ref.	0.198
High	222	0.88 (0.65–1.20)		0.94(0.69–1.29)		0.85(0.66–1.10)	19	0.84(0.65–1.10)	
*CYP2C9*									
Low	223	Ref.		Ref.		Ref.		Ref.	
High	222	0.81 (0.59–1.09)	0.165	0.81 (0.60–1.11)	0.194	0.92 (0.71–1.19)	0.523	0.96 (0.75–1.25)	0.774
*CYP2C18*									
Low	223	Ref.	0.502	Ref.	0.561	Ref.	0.954	Ref.	0.945
High	222	0.90 (0.66–1.22)		0.91 (0.67–1.24)		0.99 (0.77–1.28)		1.01 (0.78–1.31)	
*CYP2C19*									
Low	223	Ref.	0.605	Ref.	0.460	Ref.	0.826	Ref.	0.850
High	222	0.92 (0.68–1.25)		0.89 (0.65–1.21)		0.97 (0.75–1.25)		0.98 (0.75–1.26)	

a Adjusted *P*, adjustment of sex, age, HBV–virus status, ALT, main tumor size, multinodular, cirrhosis, AFP and BCLC stage.

**Figure 4:**
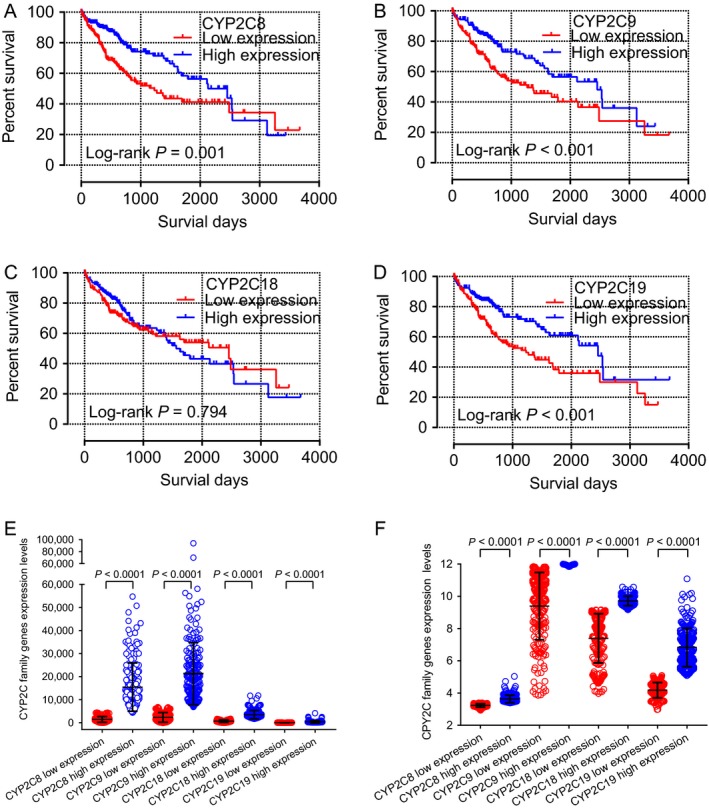
Kaplan–Meier survival curves of the *CYP2C8* (A), *CYP2C9* (B), *CYP2C18* (C), and *CYP2C19* (D) genes in the TCGA database. Scatter plots of *CYP2C8*,*CYP2C9*,*CYP2C18*, and *CYP2C19* genes expression levels in the TCGA database (E) and GEO database (F).

As shown by the survival curves of CYP2C8, CYP2C9, CYP2C18, and CYP2C19, based on data retrieved from the TCGA database, which are presented in Figure 4A–D, *CYP2C8*,* CYP2C9*, and *CYP2C19* were significantly associated with survival (*P* = 0.001, <0.001, and <0.001, respectively). However, survival curves of these genes, based on data retrieved from the GEO database, as presented in Figure 4A–H, showed that none were significantly associated with OS or RFS (all *P* > 0.05). In addition, scatter diagrams of the expression levels of these genes, based on data retrieved from both databases, are presented in Figure 4E and F.

### Joint effects analysis of CYP2C subfamily members

**Figure 5:**
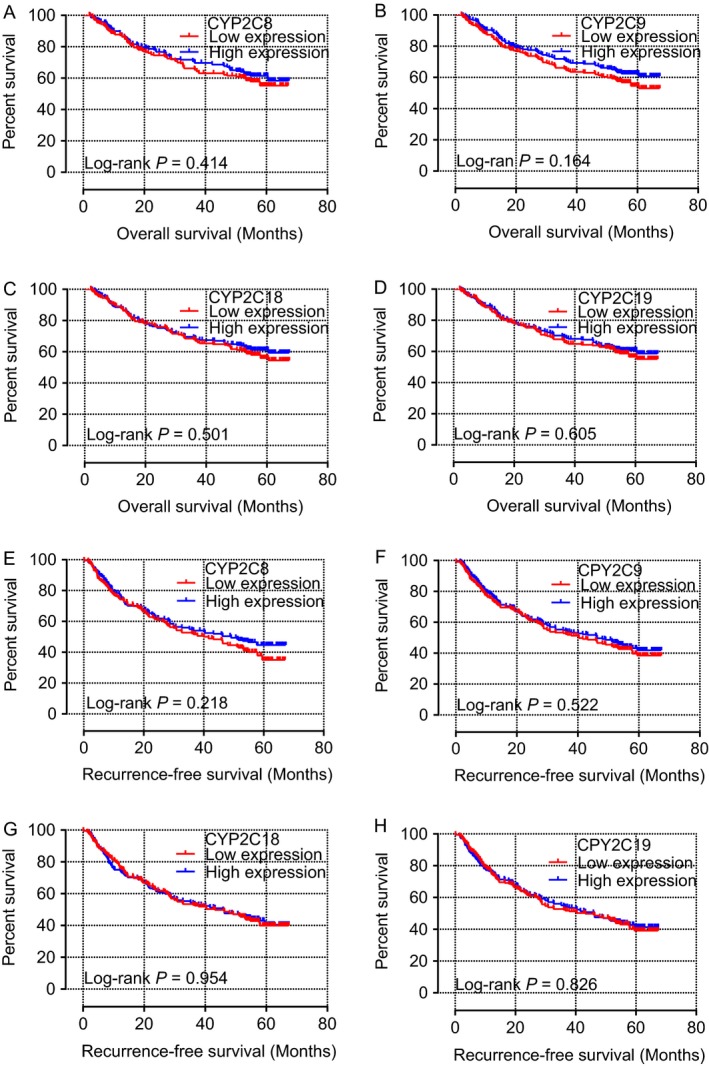
Kaplan–Meier overall survival curves of *CYP2C8* (A), *CYP2C9* (B), *CYP2C18* (C), and *CYP2C19* (D), as well as recurrence‐free survival of *CYP2C8* (E), *CYP2C9* (F), *CYP2C18* (G), and *CYP2C19* (H) in the GEO database.

Joint effects analysis of the *CYP2C8* and *CYP2C9* combination showed that MST was poorest in group 1 (931 days; adjusted *P* = 0.031) and best in group 4 (2456 days; adjusted *P* = 0.005). Meanwhile, analysis of the *CYP2C8* and *CYP2C19* combination showed that MST was poorest in group I (899 days; adjusted *P* = 0.005) and best in group IV (2456 days; adjusted *P* = 0.013), and that of the *CYP2C9* and *CYP2C19* combination showed the poorest MST in group **a** (1005 days; adjusted *b* = 0.097) and the best in group **d** (2456 days; adjusted *P* = 0.016). Detailed joint effects analysis results are shown in Table 5 and associated survival curves are shown in Figure 5A–C.

**Table 5:** Joint effects analysis of the combinations of *CYP2C8* and *CYP2C9; CYP2C8* and *CYP2C19; CYP2C9* and *CYP2C19* genes

Group	*CYP2C8* expression	*CYP2C9* expression	*CYP2C19* expression	Patients (*n* = 360)	MST (days)	Crude HR (95% CI)	Crude *P* value	Adjusted HR (95% CI)^a^	Adjusted *P* value^a^
1	Low	Low		126	931	Ref.	**0.002**	Ref.	**0.031**
2	Low	High		54	1694	0.61 (0.36–1.04)	0.071	0.82 (0.46–1.44)	0.483
3	High	Low		54	1791	0.60 (0.35–1.03)	0.064	0.61 (0.33–1.10)	0.102
4	High	High		126	2456	0.44 (0.29–0.67)	**<0.001**	0.51 (0.32–0.81)	**0.005**
I	Low		Low	123	899	Ref.	**<0.001**	Ref.	**0.005**
II	Low		High	57	NA	0.52 (0.30–0.90)	**0.020**	0.80 (0.50–1.29)	0.356
III	High		Low	57	1685	0.54 (0.32–0.92)	**0.023**	0.24 (0.10–0.61)	**0.003**
IV	High		High	123	2456	0.43 (0.28–0.66)	**<0.001**	0.54 (0.34–0.88)	**0.013**
a		Low	Low	144	1005	Ref.	**0.003**	Ref.	0.097
b		Low	High	36	NA	0.54 (0.27–1.08)	0.082	0.63 (0.31–1.28)	0.200
c		High	Low	36	1694	0.60 (0.32–1.12)	0.109	0.75 (0.39–1.42)	0.374
d		High	High	144	2456	0.49 (0.33–0.72)	**<0.001**	0.58 (0.37–0.90)	**0.016**

a Adjusted *P*, adjustment for sex, age, TNM stage. The significance is that all the values are statistically significant.

**Figure 6:**
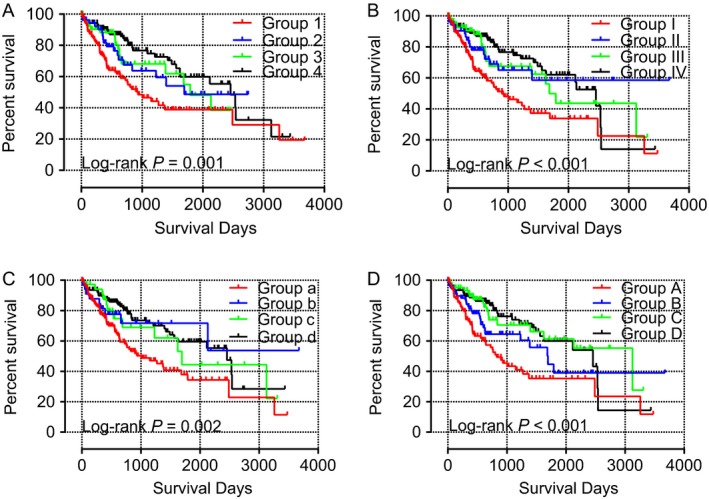
Survival curves of the joint effects analysis of the combination of *CYP2C8* and *CYP2C9* (A), *CYP2C8* and *CYP2C19* (B), *CYP2C9* and *CYP2C19* (C), and *CYP2C8*,*CYP2C9*, and *CYP2C19* (D) in the TCGA database.

Finally, joint effects analysis of the *CYP2C8*,* CYP2C9*, and *CYP2C19* combinations showed that MST was poorest in group A (827 days; adjusted *P* = 0.017) and best in group C (3125 days; adjusted *P* = 0.012). Surprisingly, MST could not be determined for group D, which contained the best factors for patients, possibly due to the influence of other potential elements (Table 6). Survival curves of the above analysis are presented in Figure 6D.

**Table 6:** Joint effects analysis of the combination of *CYP2C8*,* CYP2C9,* and *CYP2C19* genes

Group	*CYP2C8* expression	*CYP2C9* expression	*CYP2C19* expression	Patients (*n *= 360)	MST (days)	Crude HR (95% CI)	Crude *P* value	Adjusted HR (95% CI)^a^	Adjusted *P* value^a^
A	Low	Low	Low	103	827	Ref.	**<0.001**	Ref.	**0.017**
B	Low	Low	High	84	1694	0.66 (0.42–1.05)	0.080	0.77 (0.47–1.26)	0.298
	Low	High	Low						
	High	Low	Low						
C	High	High	Low	63	3125	0.40 (0.23–0.69)	**0.001**	0.47 (0.27–0.85)	**0.012**
	Low	High	High						
	High	Low	High						
D	High	High	High	110	2456	0.42(0.27–0.66)	**<0.001**	0.51(0.31–0.84)	**0.008**

a Adjusted *P*, adjustment for sex, age, TNM stage. The significance is that all the values are statistically significant.

## Discussion

In this study, the associations between CYP2C subfamily genes with HCC were investigated in both TCGA and GEO databases. The results showed that low gene expression levels of *CYP2C8*,* CYP2C9,* and *CYP2C19* in TCGA database were associated with poor prognosis of HCC. Moreover, the groups, in TCGA database analysis, with the most poor prognostic factors had the poorest prognosis in the combination analysis of the above three genes. Thus, gene expression levels of *CYP2C8*,* CYP2C9*, and *CYP2C19*—in TCGA database— both alone and in combination, may serve as potential biomarkers of HCC.

CYP2C subfamily members participate in the metabolism of many endogenous and exogenous substances. It is estimated that approximately 30% of all drugs are metabolized by *CYP2C8*,* CYP2C9*,* CYP2C18*, and *CYP2C19* 27. Moreover, *CYP2C9*,* CYP2C19*, and *CYP2C8* contribute to 17%, 10%, and 6% of drug biotransformations, respectively 28. Specifically, *CYP2C8* is reported to metabolize analgesics 29 as well as antidiabetics and cholesterol‐lowering drugs 30, while *CYP2C9* was found to metabolize analgesics 31 and neurological drugs 32, and *CYP2C19* has been linked to the metabolism of antidepressants and antipsychotics 33, as well as drugs for treatment of respiratory diseases and allergies 34. Among them, *CYP2C18* has been less well studied. Furthermore, members of the CYP2C subfamily have been implicated in drug metabolism and have also been explored in many diseases, including several cancers. Specifically, genetic variants of *CYP2C8* have been associated with an increased risk of myocardial infarction 35, paclitaxel‐induced neuropathy 36, and bisphosphonate‐related osteonecrosis of the jaw in multiple myeloma 37 and esophageal squamous cell carcinoma 38. A *CYP2C9* gene polymorphism has been associated with increased susceptibility to colorectal cancer and adenoma 39, increased progression of nonalcoholic fatty liver disease 40, and excessive anticoagulation and bleeding risk in patients taking warfarin 41. Also, mutant alleles of *CYP2C18* have been linked to *CYP2C19* in a Japanese population 42. Genetic polymorphisms of *CYP2C19* were found to be associated with a greater risk of HCC in Japanese cirrhotic patients with HCV infection 43, as well as a significant risk of triple‐negative breast cancer 44 and lung cancer in combination analysis with smoking in a Chinese population 45.

CYP2C subfamily members are highly expressed in normal liver tissue and mainly metabolize endogenous and exogenous substances as well as clinical drugs. A previous study reported that CYP2C subfamily members in human hepatocytes were affected by different inflammatory cytokines, including bacterial lipopolysaccharide, interleukin 6, tumor necrosis factor–*α*, interferon *γ*, transforming growth factor *β*, and interleukin 1*β*. Meanwhile, with regard to the four members, *CYP2C8* was downregulated by each of the above elements, *CYP2C9* and *CYP2C19*, which had almost identical response patterns, gave rise to cytokine‐specific outcomes. However, *CYP2C18* was not affected by any treatment 46. Moreover, CYP2C subfamily members are involved in the metabolic pathways of arachidonic acid, linoleic acid, retinol, as well as drug metabolism of cytochrome P450, serotonergic synapses, and chemical carcinogenesis.

In chemical carcinogenesis metabolism, benzo[a]pyrene can be metabolized by *CYP2C8*,* CYP2C9*,* CYP2C18*, and *CYP2C19*, and then finally transformed into the DNA adduct (+)‐trans‐BPDE‐N_2_‐dG, which has been shown to promote cancers of the skin, lung, and stomach. In addition, the *CYP2C8*,* CYP2C9*,* CYP2C18*, and *CYP2C19* genes are linked to *CYP1A2* in physical interactions, co‐expression, shared protein domains, co‐localization, other various pathways, and even predicted relationships. At the protein–protein interaction level, CYP2C8, CYP2C9, CYP2C18, and CYP2C19 were related to CYP1A1 and CYP1A2 in coexpression, protein homology, text mining, predicted gene neighborhood interactions, predicted gene fusions interactions, predicted gene co‐expression interactions, and other known interactions, as noted in curated databases and as determined experimentally.

These results further confirmed that CYP2C subfamily members exhibit many interactions with *CYP1A1* and *CYP1A2*. *CYP1A1* is known to participate in the metabolism of Sudan I to 8‐(phenylazo)guanine in DNA, 1, 2‐naphthoquinone, 3′,4′–diOH–Sudan I, and 4′,6′ –diOH–Sudan I, as well as DNA, RNA, and protein adducts. Among them, 8‐(phenylazo)guanine in DNA and DNA, RNA, and protein adducts can result in cancers of the liver and bladder. Meanwhile, *CYP1A2* can metabolize IQ and MeIQx and finally into DNA adducts (dG‐C8‐MeIQx, dG‐N‐MeIQx). The above DNA adducts can lead to tumorigenesis in cancers of the liver, lung, colon, and breast. In view of these results, *CYP2C8*,* CYP2C9*,* CYP2C18*, and *CYP2C19* may be associated with the occurrence of HCC. Therefore, *CYP2C8*,* CYP2C9*,* CYP2C18*, and *CYP2C19* may serve as potential diagnostic and prognostic serum biomarkers for HCC diagnosis.

It is well‐known that serum AFP is the most widely used biomarker for early diagnosis and monitoring of HCC recurrence 47. However, the prognostic value of AFP remains controversial. Several studies refuted the prognostic value of AFP in single, small HCC, and even for the prediction of HCC recurrence 48, 49. Several literatures reported its sensitivity of less than 70% at a cutoff value of 20 ng/mL 50, 51.

Many novel serum biomarkers of HCC have been identified in recent years, including osteopontin 52, UQCRH 53, CXCL1 54, integrator complex subunit 6 55, PIVKA–II 56, TIP 30 57, cavin–2 58, and annexin A2 59, among others. Although a variety of potential serum biomarkers were put forward by different research centers, clinical applications have been limited because of the highly heterogeneous nature of HCC. In the present population, CYP2C subfamily gene expression levels were associated with HCC prognosis. Thus, we postulate that the CYP2C subfamily members may serve as potential serum biomarkers for the early diagnosis of HCC.

However, there were some limitations in this study. First, larger population studies are required to increase the credibility of these conclusions. Second, other potential influencing factors regarding tumor evolution and prognosis, such as drinking status, smoking status, cirrhosis status, Child–Pugh score, tumor number, primary tumor size, pathological differentiation diagnosis, tumor capsule status, and vascular invasion should be included for analysis to better evaluate the relationships between CYP2C subfamily members and HCC prognosis. Third, more commonly used indicators, such as disease‐free survival, should be considered to estimate HCC prognosis. Fourth, further well‐designed studies concentrating on functional validation are warranted with a greater number of research centers and more racially diverse countries. Fifth, other significant drug‐metabolizing CYPs, including CYP1A2, CYP2A6, CYP2B6, CYP2D6, CYP2E1, and CYP3A4/5, will be explored for HCC in our future studies. To summarize, the results of this study indicate that *CYP2C8*,* CYP2C9*, and *CYP2C19* present potential serum biomarkers for the early diagnosis of HCC and combination analysis showed significant interactions that were better prognostic indicators of HCC. However, because of the incomplete clinical data and small sample size in this study, further research is necessary to validate these findings.

## Conflict of Interest

No conflicts of interest were disclosed in this study.

## Supporting information


**Figure S1**. Expression levels of *CYP2C8*,* CYP2C9*,* CYP2C18,* and *CYP2C19* genes in different tissues.
