# Supplementary figures and images for: The prognostic value of CYP2C subfamily genes in hepatocellular carcinoma
Source: Cancer Med. 2018 Feb 26;7(4):966–80. doi: 10.1002/cam4.1299 (PMC5911570; doi:10.1002/cam4.1299)

A

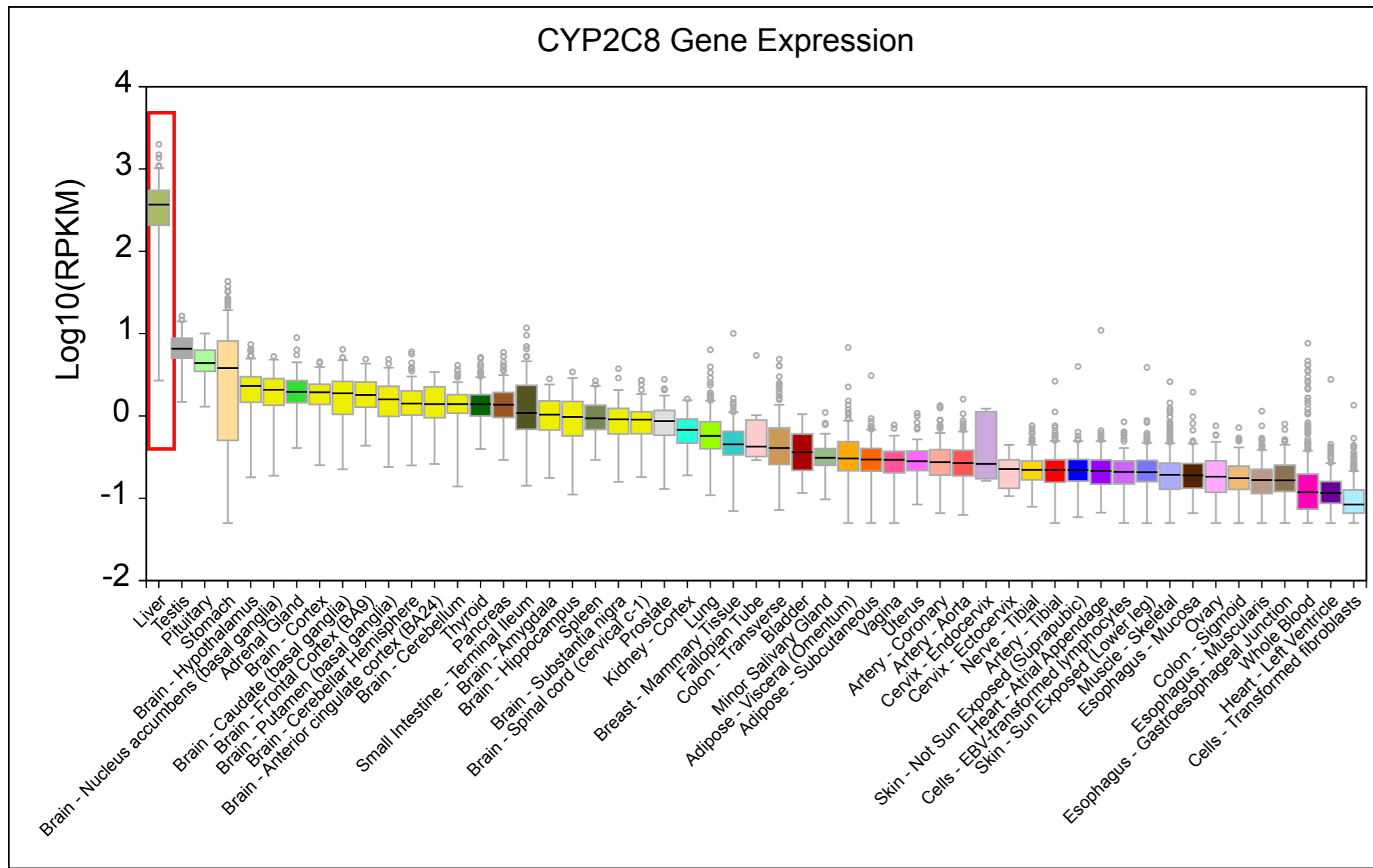

B

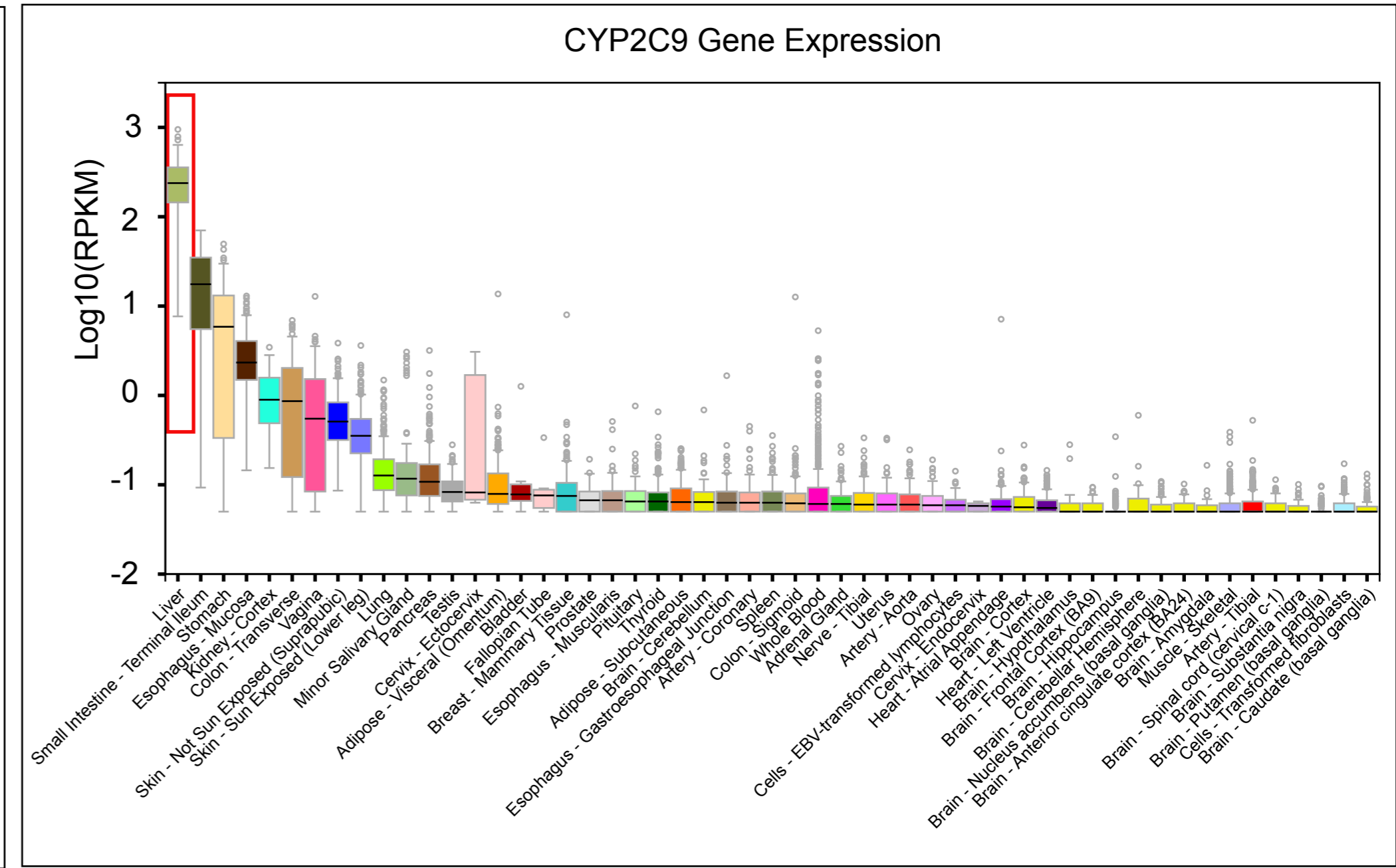

C

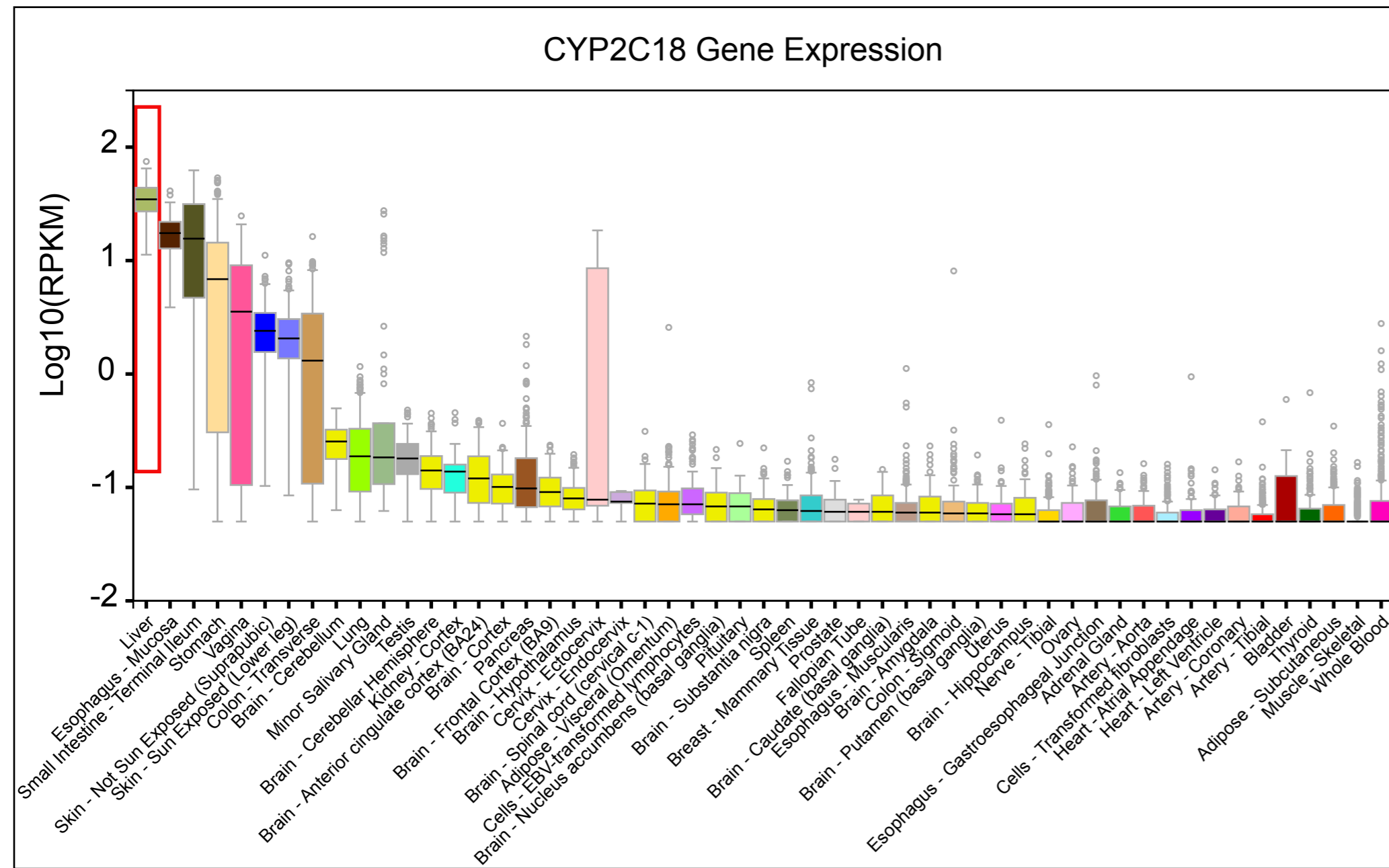

D

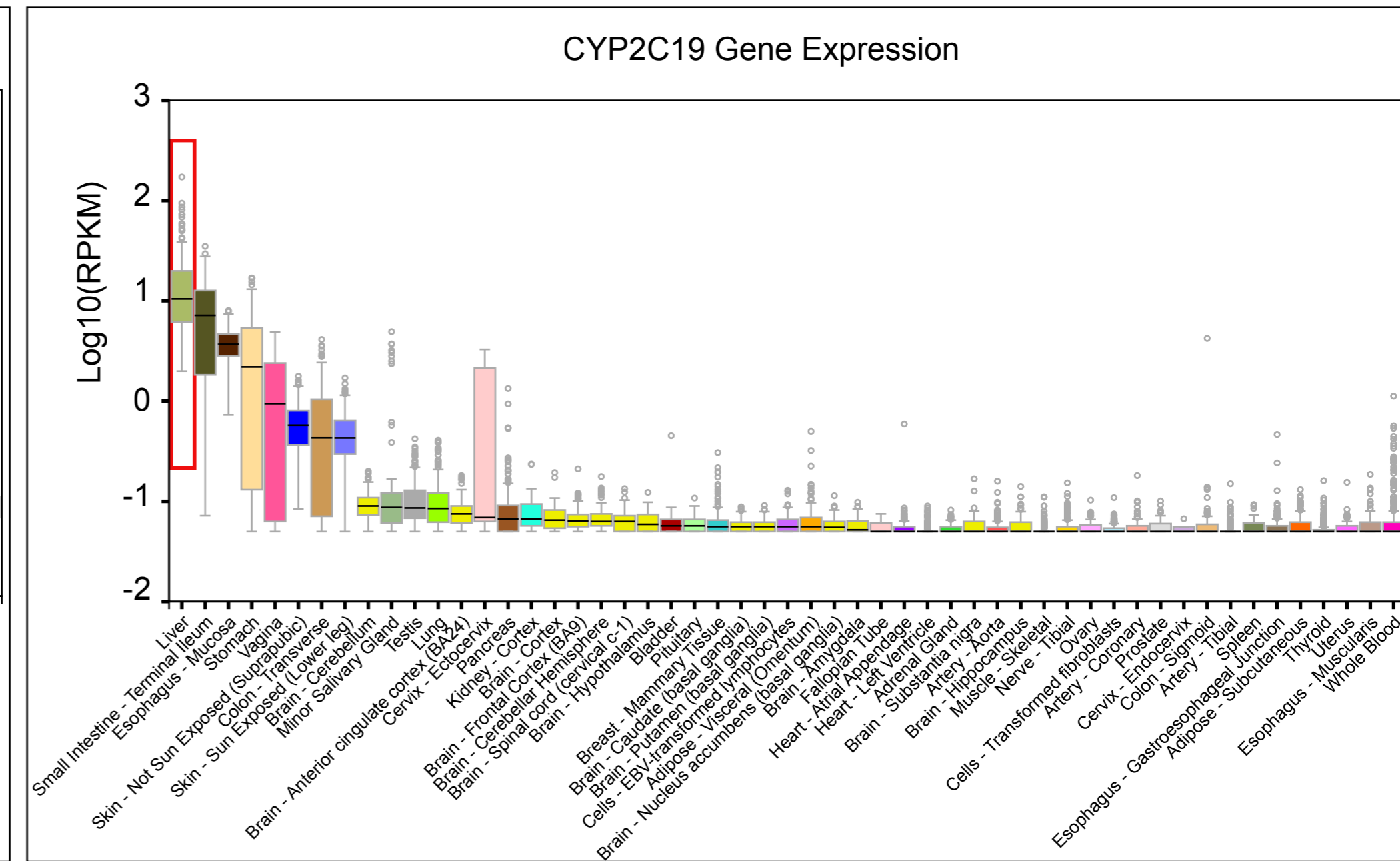

Supplement: Supplementary file 1 — Figure S1. Expression levels of CYP2C8, CYP2C9, CYP2C18, and CYP2C19 genes in different tissues. [file CAM4-7-966-s001.pdf]
